# Recurrent allopolyploidization, Y-chromosome introgression and the evolution of sexual systems in the plant genus *Mercurialis*

**DOI:** 10.1098/rstb.2021.0224

**Published:** 2022-05-09

**Authors:** J. F. Gerchen, P. Veltsos, J. R. Pannell

**Affiliations:** Department of Ecology and Evolution, University of Lausanne, 1015 Lausanne, Switzerland

**Keywords:** sex chromosome, phylogenetics, androdioecy

## Abstract

The plant genus *Mercurialis* includes dioecious, monoecious and androdioecious species (where males coexist with hermaphrodites). Its diversification involved reticulate evolution via hybridization and polyploidization. The Y chromosome of the diploid species *Mercurialis annua* shows only mild signs of degeneration. We used sequence variation at a Y-linked locus in several species and at multiple autosomal and pseudoautosomal loci to investigate the origin and evolution of the Y chromosome across the genus. Our study provides evidence for further cases of allopolyploid speciation. It also reveals that all lineages with separate sexes (with one possible exception) share the same ancestral Y chromosome. Surprisingly, males in androdioecious populations of hexaploid *M. annua* carry a Y chromosome that is not derived from either of its two putative progenitor lineages but from a more distantly related perennial dioecious lineage via introgression. These results throw new light on the evolution of sexual systems and polyploidy in *Mercurialis* and secure it as a promising model for further study of plant sex chromosomes.

This article is part of the theme issue ‘Sex determination and sex chromosome evolution in land plants’.

## 1. Introduction

Dioecious plants provide valuable material for studying the earliest stages of sex-chromosome evolution [1,2]. Because dioecy is usually derived from hermaphroditism, their sex chromosomes must postdate that transition and may thus often be relatively young. In contrast to animals, in which separate sexes are ancestral and conserved and the same genes are thus often involved in determining sex (reviewed in [3]), the genetic details of sex determination in plants tend to differ among species [4–8]. The convergent evolution of dioecy in flowering plants thus offers opportunities for understanding not only why and how separate sexes evolve but also how sex determination and sex chromosomes evolve in different independent lineages.

Populations or species with sexual system variation point to particularly recent evolutionary transitions that allow close comparisons of sex chromosomes in different contexts. Several such cases have been studied in some detail. The quantitative dimensions of sex allocation, including reversions from dioecy to hermaphroditism, were explored by Lloyd [9] in the genus *Leptinella* (syn. *Cotula*), which shows variation among lineages between combined and separate sexes. Important insights have also been gained from other systems with similar sexual-system variation (e.g. *Wurmbea* [10,11], *Sagittaria* [12,13], *Ecballium* [14,15] and *Mercurialis* [16,17]). Among-species variation in sex determination has been studied in the poplar and willow family (Salicaceae) and in wild strawberries, *Fragaria*. In the Salicaceae, sex-chromosome evolution has involved the independent adoption of homologues of a single gene with a role in sex determination in different species [7,18,19], as in some animal groups. In octoploid *Fragaria*, femaleness is determined by a cassette of genes whose chromosomal position differs between species, probably owing to translocations between homeologous chromosomes [20], and with the continued maintenance of separate sexes during diversification. Dioecy was also probably conserved during divergence of two closely related lineages of *Rumex hastatulus*, where one lineage retained an ancestral XY sex determination system and the other acquired a second Y chromosome through an X-autosome fusion [21–23].

Most work on sexual system transitions and the evolution of sex chromosomes has concerned the evolution of dioecy from hermaphroditism, which was long regarded as an evolutionary dead end [24]. However, it is now clear that reversions to hermaphroditism have been frequent [25,26], probably driven by selection of self-fertile hermaphrodites that confer reproductive assurance [27–29]. Something like this is likely to have occurred in *Vitis*, where hermaphrodites probably resulted from recombination between male- and female-sterility loci in a sex-determining region and domestication that favoured uniparental reproduction [30]. Similarly, cultivated *Carica papaya* is hermaphroditic, with a modified Y chromosome, whereas wild populations are dioecious [31]. In a process not unlike domestication, experimental evolution of dioecious populations of the plant *Mercurialis annua* demonstrated a rapid transition from dioecy to hermaphroditism in just a few generations via the selection of ‘leaky' sex expression in females following the removal of males [32].

Another important question concerns the effect of polyploidy on sexual systems and sex determination [33], not least because so many angiosperms have a recent history of polyploidization [34,35]. Indeed, polyploidy is found in several plant clades in which sex chromosomes have been studied, e.g. *Salix* [36], *Silene* [37], *Rumex* [38] and *Fragaria* [20]. Genome duplication can lead to problems of meiosis and, in species in which sex-determination depends on the relative dosage of the sex-determining alleles, can cause failure of the sex-determining system, precipitating a transition from dioecy to hermaphroditism [33]. When polyploidization involves hybridization between two dioecious species, it is also pertinent to ask whether dioecy is maintained in the allopolyploid hybrid, and if so, which of the two progenitor species contributed the sex-determination locus (if indeed either of them did).

**Figure 1.**
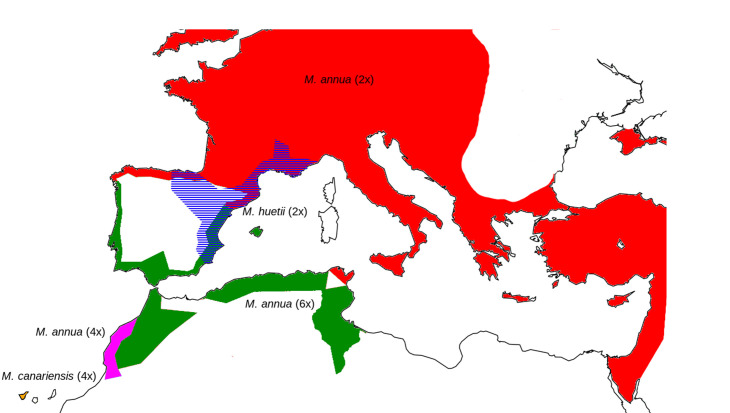
Distribution of lineages of annual *Mercurialis* species across Europe, North Africa and the Middle East. Red, diploid dioecious *Mercurialis annua*; purple, tetraploid monoecious *M. annua*; green, hexaploid androdioecious *M. annua*; orange, tetraploid dioecious *Mercurialis canarienesis*; blue, diploid dioecious *Mercurialis huetii*.

Here, we present a phylogenetic analysis of the evolution of sexual systems and sex chromosomes in the genus *Mercurialis* (Euphorbiaceae), in which sex chromosomes have been evolving in the context of transitions both between sexual systems and among ploidy levels [16,17,39]. *Mercurialis* is an almost exclusively European genus with several perennial and annual species, most of which are dioecious (table 1 and figure 1). Both pereniallity and dioecy are ancestral in the genus, and monoecy has evolved in annual species that diversified in the context of both genome duplication and hybridization [17,40]. Diploid *M. annua* has an XY sex chromosome system, and crosses among several of the annual species suggest that they all have the same sex determination system and probably the same sex chromosomes [41]. Veltsos *et al*. [42,43] studied the genome and sex chromosomes of diploid *M. annua* and found evidence for a large but only mildly degenerate sex-determining region, and Li *et al.* [44] showed that YY males (that thus lacked an X chromosome) were fully viable, though partially sterile, consistent with the low divergence between the X and Y. Our study builds on this work by asking how the *M. annua* sex chromosomes relate to those in the other *Mercurialis* species with separate sexes.

**Table 1.** *Mercurialis* lineages used in this study. (Two different chromosome counts were published for *Mercurialis elliptica*, the lower count is more likely to be correct but needs verification.)

lineage	sexual system	life history	chromosome count	ploidy
*Mercurialis annua*	dioecy	annual	16	diploid
*Mercurialis annua*	monoecy/androdioecy	annual	32	tetraploid
*Mercurialis annua*	monoecy/androdioecy	annual	48	hexaploid
*Mercurialis canariensis*	dioecy	annual	32	tetraploid
*Mercurialis huetii*	dioecy	annual	16	diploid
*Mercurialis reverchonii*	dioecy	perennial	26	tetraploid
*Mercurialis tomentosa*	dioecy	perennial	26	tetraploid
*Mercurialius elliptica*	dioecy	perennial	42 (220)	hexaploid

Our phylogenetic analysis represents an advance on those of Obbard *et al*. [17] and Ma *et al*. [40], which were based on sequence variation at nuclear internal transcribed spacer and plastid loci. By contrast, we considered the topologies of phylogenetic trees for multiple loci on both autosomes and the sex chromosomes, including a locus that amplifies only in males in most species of the genus and that thus allowed us to consider the topology of the sex-determining region itself. We were particularly interested in discovering the phylogenetic origin of androdioecy in hexaploid *M. annua* [45]. Androdioecy, a rare sexual system in both plants and animals in which males co-occur with hermaphrodites [46–48], has usually evolved from hermaphroditism via the breakdown of dioecy [28,49], but Obbard *et al*. [17] suggested that androdioecy in hexaploid *M. annua* may have evolved following hybridization between tetraploid monoecious *M. annua* and *Mercurialis huetii*, from which it was thought to have derived its Y chromosome. Our results clearly reject this hypothesis: the Y chromosome in hexaploid androdioecious *M. annua* appears to be the result of unusual sex-chromosome introgression from a more distantly related perennial species. Our analysis also points to previously unrecognized allopolyploidization in both annual and perennial *Mercurialis* species and indicates that dioecy was probably maintained through the process of polyploidization.

## 2. Material and methods

### (a) Species sampled

Table 1 lists all species sampled in our study, and figure 1 shows the present distribution of the annual species, our prime focus. For lineages with males, we included one male and either one female or one monoecious individual. For diploid and hexaploid *M. annua*, we included samples from the eastern and western ranges of their distributions. We also included two recently described hexaploid monoecious individuals with male-like inflorescences [40]. Tetraploid *M. annua* has been previously described as monoecious [17,39], but we have since found a few males in tetraploid populations south of Casablanca, and we included one of these in our dataset (electronic supplementary material, table S2).

### (b) Overview of the development of new phylogenetically informative loci

We developed 24 phylogenetically informative loci for our study. All but one of them amplified in all males, females and hermaphrodites, so that their polymerase chain reaction (PCR) products contained mixtures of multiple sequences representing alleles or diverged homeologous sequences in polyploids. The inferred chromosomal location of these loci was based on the linkage map developed by Veltsos *et al.* [42]; we refer them as either ‘autosomal' (located on one of the autosomes), ‘pseudoautosomal' (located on the sex chromosomes, but outside the region of suppressed recombination in diploid *M. annua* males) or ‘sex-linked' (located on the sex chromosomes inside the region of suppressed recombination in diploid *M. annua* males). In addition, one locus amplified in males as a single copy, regardless of ploidy, indicating tight linkage to the sex-determining region on the Y chromosome across the genus. We refer to this locus as ‘male-specific'. All loci were based on genomic regions covered by aligned exon-capture datasets independent of gene models, i.e. the amplified regions were mostly exonic (but could also include intronic or intergenic regions) and were not complete genes. Further details about the development of the loci are given in the electronic supplementary material.

### (c) Development of phylogenetically informative loci on sex chromosomes and autosomes

Development and analysis of phylogenetically informative loci in allopolyploids requires accounting for multiple homeologous or allelic copies of the same sequence that must be distinguished to infer origins of allopolyploid subgenomes [50]. We thus used long-read sequencing to obtain reads that span the complete amplified region of PCR products without the need for cloning [51]. We then phased homeologous sequences from multiple loci, based on phylogenetic information and using an iterative approach, and generated multi-locus phylogenies with these loci. The pipeline used is described in the electronic supplementary material.

### (d) Polymerase chain reaction amplification and sequencing of autosomal and sex-linked loci

We selected 24 primer pairs, 15 of which were located on the sex chromosome and one or two on each of the autosomes (electronic supplementary material, figure S3). We performed PCR with these (electronic supplementary material, table S1) for each of 24 samples of perennial and annual *Mercurialis* species (electronic supplementary material, table S2) using 1× Qiagen Hotstart PCR Buffer, 0.2 µm dNTPS, 0.2 µm forward and reverse primer and 0.001 U µl^−1^ Hotstart Taq Polymerase (Qiagen). The PCR protocol was 15 min of initial denaturation at 95°C, followed by 35 cycles of amplification with initial denaturation for 30 s at 94°C, annealing for 30 s and elongation at 73°C for 1 min, and final elongation at 73°C for 5 min.

After amplification, we pooled PCR products for each sample in approximately equimolar amounts, based on the intensity of agarose gel bands, cleaned the pools using cleanNGS beads (Labgene) according to the manufacturer's instructions and quantified DNA using a Qubit fluorometer (Fisher Scientific). In a second step, we ligated individual barcodes to each of these pools and combined pools to obtain a single barcoded Oxford Nanopore sequencing library. We followed the recommendations for native barcoding of genomic DNA using the ligation sequencing kit and the native barcoding extension 1–12 and 13–24 from Oxford Nanopore. About 100 fmol of the final library were sequenced on an Oxford Nanopore 9.4.1 flowcell. Raw reads were base-called and demultiplexed using Guppy 4.0.15 (Oxford Nanopore).

In polyploid samples, the resulting long reads represent a mixture of alleles from different homeologues. Owing to the high error rates of Oxford Nanopore reads, final alignments have to be based on a consensus of multiple reads representing unique template sequences, which requires clustering of raw reads. We developed a new approach, which clusters reads at each sample and locus using a custom Python script (02_cluster_pcr_reads; see the electronic supplementary material for details).

Our dataset could contain multiple sequences per locus for the polyploid genomes, representing fixed differences between, or allelic variation within, homeologous sequences. In a first step, we built single-locus phylogenies, which contained both types of sequences using RAxML 8.2.12 (parameters ‘-f d','-d', ‘-# 100', ‘-m GTRGAMMA’, 1000 bootstrap replicates). These single-locus phylogenies may be limited in size and phylogenetic resolution. To overcome these limitations, we also built multi-locus phylogenies. These should represent differences between homeologues and should not include the within-locus allelic variation that was removed using a custom Python script (03_subset_alignment; see the electronic supplementary material for details).

In general, polyploid sequences cannot be simply concatenated for generating multi-locus phylogenies, because the phase of homeologous sequences from different loci of the same polyploid individual is typically unknown. We sequentially phased homeologous sequences for multiple sets of loci with a custom workflow implemented in Python and Biopython 1.79 (04_multilocus_phylogeny; see the electronic supplementary material for details). We ran this analysis for the full dataset including all loci and independently for three subsets of the autosomal, pseudoautosomal and sex-linked loci.

### (e) Development of a male-specific polymerase chain reaction locus

We previously identified 17 exonic loci with Y-linked inheritance throughout the diploid *M. annua* species range, based on the published exon-capture dataset [52], and confirmed their Y linkage with PCR and Sanger sequencing [42]. We extended the PCR assay for one of these loci (g3639/gm56331, located on contig56631 of v. 1.3 of the *M. annua* genome assembly), using DNA from male and female/monoecious samples of annual and perennial lineages of the *M. annua* species complex. We then used the previously described newly developed exon-capture dataset to develop a longer sex-linked sequence at the same locus, which amplified in annual lineages as well as in perennial *Mercurialis reverchonii*, *Mercurialis tomentosa* and *Mercurialis elliptica*. Reads were aligned against v. 1.3 of the *M. annua* genome assembly using the previously described pipeline. We then generated consensus sequences based on aligned exon-capture reads at the sex-linked contig for males from annual and perennial lineages of *Mercurialis*, using a pileup from the Python implementation of Samtools 1.9 [53] and marking variable sites with Ns. We used these sequences to search for PCR primers using primer3plus [54] and developed four different primer pairs that target the same locus in different lineages (electronic supplementary material, table S1).

To test for sex-specificity of the loci, we applied them to males and females or monoecious (negative control) samples of annual and perennial *Mercurialis* species using 1× Hotstart PCR buffer (Qiagen), 0.2 μm dNTPS, 0.2 μm forward and reverse primer and 0.001 U µl^−1^ Hotstart Taq Polymerase (Qiagen). The PCR protocol was 15 min of initial denaturation at 95°C followed by 35 cycles of amplification with initial denaturation for 30 s at 94°C, annealing at 60°C for 30 s and elongation at 73°C for 1 min. Final elongation was at 73°C for 5 min. Afterwards, we visualized bands using gel electrophoresis and sent a subset of PCR products of males that amplified successfully for Sanger sequencing to Microsynth (Balgach, Switzerland).

We aligned the *Mercurialis* sequences using MAFFT 7.475 [55] and aligned each of the diploid *M. annua* sequences against the NCBI nucleotide collection [56] using blastn [57] to identify the best unique outgroup sequence. We added the sequence of the resulting best BLAST hit to the alignment, realigned the sequences using MAFFT and generated a phylogenetic tree using RAxML 8.2.12 (parameters ‘-m GTR' and 100 bootstrap replicates) [58].

## 3. Results

### (a) Phylogenetic inference based on the autosomal and sex-chromosome loci

We generated a total 2.2 Gb of Oxford nanopore long-reads from PCR amplicons of the newly developed phylogenetically informative loci from autosomal and sex-linked loci (electronic supplementary material, table S1 and figure S3). Upon inspection of alignments of demultiplexed reads to the *M. annua* genome, we noted that a significant proportion of reads for primer pair 1_24 were much shorter than expected, based on the location of primers at the *M. annua* genome assembly. We interpreted this as a result of non-specific PCR amplification and excluded this locus from further analyses. For the remaining loci, we excluded a total of 13 (2.4%) low-coverage samples that had a sequencing depth smaller than 10 times the ploidy at the target locus. The full concatenated and phased alignment, which included all 23 remaining loci, was 22 928 bp long.

**Figure 2.**
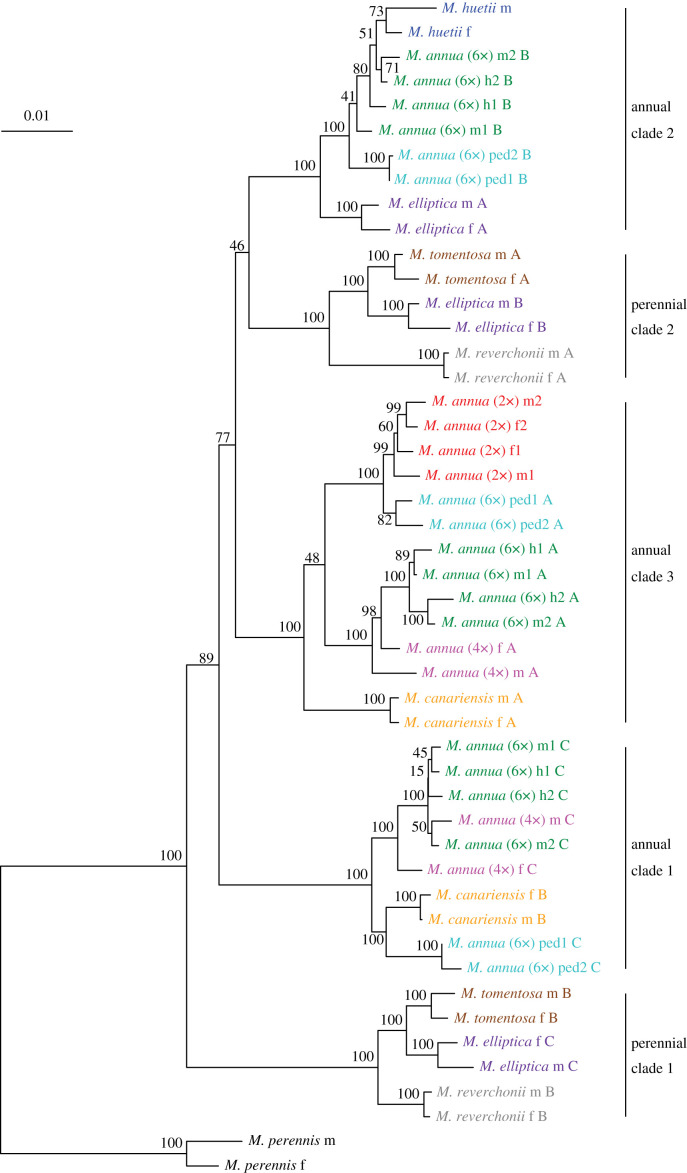
Multi-locus phylogeny based on 23 phylogenetically informative loci located on the sex chromosomes and autosomes. Values next to nodes indicate bootstrap support. The scale bar indicates the substitution rate. m, f, h and ped at the end of species names at the tips describe male, female, monoecious individuals and monoecious individuals with male-like inflorescences, respectively. A, B and C indicate different homeologous sequences from the same polyploid individual.

The phylogenetic tree resulting from analysis of the autosomal, pseudoautosomal and sex-linked sequences includes five major monophyletic clades with a bootstrap support of 100, each of which contained homeologous sequences from multiple polyploid species (figure 2). The most basal clade relative to the *M. perennis* outgroup contains sequences from males and females from the three perennial lineages *M. reverchonii*, *M. elliptica* and *M. tomentosa* (perennial clade 1). Annual clade 1 contains sequences from each of the tetraploid and hexaploid *M. annua* lineages as well as from *Mercurialis canariensis*. In addition, perennial clade 2 contains sequences from all samples from the perennial lineages. Annual clade 2 contains sequences from the male and female *M. huetii* samples and all hexaploid *M. annua* individuals as well as sequences from one male and female perennial *M. elliptica*. Annual clade 3 contains sequences from all *M. annua* individuals of either sex and ploidy level (including the newly described monoecious hexaploids with male-like inflorescences; [40]), as well as from both male and female *M. canariensis* samples (figure 2).

All five clades were well-supported in the phylogenetic trees, based on the three subsets of the full dataset, i.e. autosomal, pseudoautosomal and sex-linked loci (electronic supplementary material, figures S4–S29), with the exception of annual clade 3 in the autosomal set of loci, which did not contain sequences of male and female *M. canariensis*; these were placed as a sister group to annual clade 1. Our analysis is consistent with that of Obbard *et al*. [17] in a number of respects, not least in the inference that both dioecy and perenniality are ancestral.

Homeologous sequences from all polyploid species are found in multiple monophyletic clades, implying an allopolyploid origin for all of them. The result confirms inferences by Obbard *et al*. [17] and implies additional allopolyploidization events. Obbard *et al*. [17] inferred that tetraploid *M. annua* was an autopolyploid, but the presence of its sequences in both annual clade 3 and annual clade 1 suggest that it is allopolyploid. In addition, the two hexaploid lineages of *M. annua* are both allopolyploid, each with three progenitors, one from each of the three annual clades. This contrasts with Obbard *et al*.'s [17] inference that all hexaploid populations were the result of hybridization between an ancestor of the inferred autotetraploid *M. annua* and an ancestor of diploid *M. huetii*. Finally, sequences of all three perennial lineages are found in diverged clades and are thus allopolyploids too. A single sequence of *M. elliptica* was found in annual clade 2, also indicating allopolyploidy.

While the lineages contained in five major clades could be clearly defined, the phylogenies based on subsets of data showed some differences in the relationships among clades. Relationships among clades were the same for the pseudoautosomal and full datasets (figure 2; electronic supplementary material, figure S5). In the dataset based on autosomal loci, the placement of annual clade 2, perennial clade 2 and annual clade 1 differed from the full dataset (electronic supplementary material, figure S4). Here, annual clade 2 and annual clade 3 formed a sister clade, while the perennial clade 2 and annual clade 3 formed a sister clade in the full dataset. Analysis of the sex-linked loci pointed to two monophyletic clades (electronic supplementary material, figure S6), one with the perennial species and annual clade 2, and the other with annual clade 1 and annual clade 3. Uncertainties in the relative placement of these clades are reflected in the low bootstrap support at some of the deeper nodes in the phylogenetic trees.

### (b) Phylogenetic inference based on the male-specific locus

**Figure 3.**
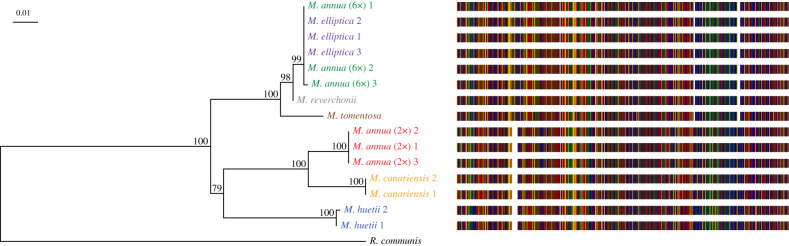
Phylogeny and alignment for the Y-specific PCR locus. Values next to nodes of the tree indicate bootstrap support, and the scale bar indicates the substitution rate. Bars on the right side of the tip labels represent the multiple sequence alignment for each sequence, with coloured bars representing nucleotides (red, A; blue, T; orange, C; green, G) and white areas indicating deletions.

The previously described male-specific locus for diploid *M. annua* was successfully amplified in a male-specific fashion in diploid dioecious *M. annua* and *M. huetii*, tetraploid dioecious *M. canariensis* and hexaploid androdioecious *M. annua* (electronic supplementary material, figure S1). This is, to our knowledge, the first evidence of a common Y chromosome in the *M. annua* species complex. Analysis of the longer sequence (707 bp) at the same locus also showed Y-specific amplification among most species with males (figure 3). We could not amplify the locus in the rare males in tetraploid *M. annua*, nor in monoecious individuals with pedunculate inflorescences of hexaploid *M. annua*, which Ma *et al*. [40] found was phylogenetically distinct from androdioecious hexaploid *M. annua*.

Alignment of the male-specific sequences revealed two deletions and one insertion that were specific to hexaploid *M. annua*, *M. reverchonii*, *M. elliptica* and *M. tomentosa* but absent in the other annual lineages. The best BLAST hit (*E* = 1 × 10^−117^, 73% identity) of the male-specific *Mercurialis* sequence was a messenger RNA (XM_002528314.3) from *Ricinus communis*, with its function predicted as serine-rich adhesin for platelets. Consistent with the pattern of indels shown in figure 3, the tree based on the male-specific locus placed hexaploid *M. annua* into a clade with the perennial *Mercurialis* species, with *M. elliptica* its closest inferred relative, while the other annual lineages formed a distinct clade (figure 3). This topology differs from that inferred on the basis of all other loci (figure 2), in which the hexaploid lineage is absent from both of the two main perennial clades (which include sequences from all three perennial lineages). Together, these results imply that the part of the Y chromosome of hexaploid *M. annua* must have been acquired through introgression from a lineage in the perennial clade, probably a close ancestor of *M. elliptica*. Significantly, the autosomal and pseudoautosomal loci, including the six sex-linked loci, shared a similar topology (electronic supplementary material, figures S5, S6 and S15–S20), indicating that only a small part of the Y chromosome including the sex-determining locus was introgressed.

## 4. Discussion

**Figure 4.**
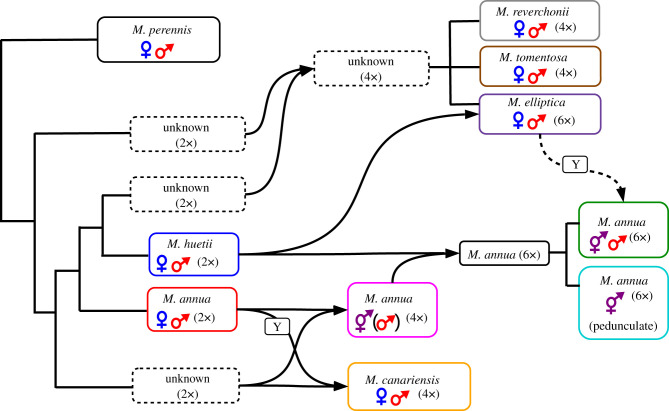
Model for the evolution of polyploid *Mercurialis* lineages. Arrows indicate polyploidization events, and dashed arrow indicates Y chromosome introgression. Lines indicate homoploid speciation events.

Our phylogenetic analyses of *Mercurialis*, based on sequence variation at 24 autosomal and sex-linked loci (one tightly linked to the sex-determination locus), suggest the evolutionary scenario summarized in figure 4. This scenario: (i) points to additional allopolyploidization events in the genus, permitting a more nuanced assessment of the relationship between sexual systems and ploidy; and (ii) allows both the identification of the source of the Y chromosome in two of the annual allopolyploid species as well as a phylogenetic explanation for the origin of androdioecy in hexaploid *M. annua*.

### (a) The robustness of our approach for phylogenetic inference

To distinguish sequences at the same locus that represent informative differences between homeologues and those that represent differences between alleles deal with the substantial challenges of phylogeny reconstruction involving allopolyploids [50], we assumed that divergence between allelic sequences should be smaller than between homeologous sequences and that the number of expected homeologous sequences should be equal to half the sample ploidy. When comparing the divergence between sequence pairs for putative allelic sequences and homeologous sequences in our dataset, there was not always a complete separation between both sequence types (electronic supplementary material, figure S30). This could result from natural variation in the divergence between homeologous sequences across loci and individuals. However, if sequences representing one homeologue were missing from the dataset, allelic sequences could be mistaken for homeologues, which could have strong effects on the inference of phylogenies and the phasing of homeologous sequences based on them. Such dropout of homeologous sequences could occur if variation in the prime region at the target locus were in one of the homeologues, or in cases of gene conversion or structural variation between homeologues. Such a possibility seems unlikely to have affected our analysis, given the generally well-supported separation of homeologous sequences into discrete clades (electronic supplementary material, figure S4–S6). Only in the autosomal dataset did putative homeologous sequences for *M. canariensis* show a pattern incongruent with these well-defined clades (electronic supplementary material, figure S4).

### (b) Allopolyploidy and sexual-system variation in *Mercurialis*

Our study confirms that a transition from a perennial to an annual life history preceded the transition among the annual species from dioecy to monoecy. It seems likely that this transition to monoecy coincided with the loss of the Y chromosome and the evolution in females of a male function in response to selection favouring enhanced ‘leaky' sex expression [27,29], which remains common in dioecious *M. annua* [59,60]. Our analyses also confirm an allopolyploid origin for both hexaploid androdioecious *M. annua* involving the diploid *M. annua* and *M. huetii* lineages and tetraploid *M. canariensis* (involving the diploid *M. annua* and an unknown lineage) and indicate that tetraploid *M. annua* is an allopolyploid (and not an autopolyploid as suggested by Obbard *et al*. [17]), that tetraploid dioecious perennial *M. tomentosa* and *M. reverchonii* are allopolyploids with two divergent genomes and that *M. elliptica* is an allohexaploid with an additional genome sharing ancestry with both diploid *M. huetii* and hexaploid androdioecious *M. annua*.

Allopolyploidy has clearly played a major role in the diversification of *Mercurialis* but has not impacted sexual-system transitions in a straightforward way. Unlike the transition from diploidy to allotetraploidy in *M. annua*, which coincided with a transition to monoecy, none of the other polyploidization events resulted in the evolution of monoecy. Dioecy may have broken down with polyploidization in these lineages and then re-evolved, but this seems unlikely. For most of the allopolyploid lineages (apart from hexaploid *M. annua*, which we discuss below), the topology for the Y-linked locus is congruent with that for the autosomal and pseudoautosomal loci used, allowing the parsimonious inference that the Y chromosome (and thus dioecy) was maintained through the ploidy transition.

Despite the loose association between ploidy and sexual system among annual species of *Mercurialis* (dioecious diploid species *M. annua* and *M. huetii*, and the largely monoecious tetraploid and hexaploid lineages of *M. annua*; [16,39]), there would appear to be no meaningful constraint linking these two traits at the genus level, and it is more reasonable to seek a functional explanation for the evolution of monoecy in polyploid *M. annua*. The fact that strong selection on sex allocation in experimental populations of diploid *M. annua* could bring about a transition from dioecy to monoecy in just a few generations [32] is consistent with this view: monoecy can just as well evolve in diploids. In angiosperms more generally, there is also no clear association between the sexual system and ploidy, with polyploidy associated with both transitions to separate sexes and combined sexes (reviewed in [33]).

Finally, it is clear that the X and Y chromosomes are not only shared among the annual species of *Mercurialis*, as inferred from simple between-species crosses by Russell & Pannell [41], but also that the same sex chromosomes are shared with perennial species *M. tomentosa*, *M. reverchonii* and *M. elliptica*. The ancestral XY system has thus evidently been conserved during the genus' diversification and, significantly, there is no evidence of sex-chromosome turnover in *Mercurialis*. This finding sets a foundation for a comparative analysis of the same Y chromosome that has been evolving independently among different species.

### (c) The origin of the Y chromosome in allopolyploid species of *Mercurialis*

Our study allows us to infer which progenitor species contributed the Y chromosome to the allopolyploid species that have retained or regained separate sexes in *Mercurialis*. It is now clear that dioecious tetraploid *M. canariensis* derives its Y chromosome from the diploid *M. annua* lineage, the most widespread lineage of the genus with a distribution across much of Europe and around the Mediterranean Basin [39,61]. *Mercurialis canariensis* has only been sampled on the island of Tenerife several thousand kilometres from the nearest populations of its one extant progenitor, diploid *M. annua* in northern Spain [62]. Nevertheless, phylogeographical inference based on gradients in genetic diversity across Europe point to a dynamic history of migrations, with *M. annua* having occupied much of Central and Western Europe from a refugium in the eastern Mediterranean, perhaps in present-day Turkey or the Middle East [63]. The range expansion into Western Europe was likely post-Pleistocene, so that *M. annua* probably occupied Northwest Africa much earlier, when *M. canariensis* likely originated. The same reasoning applies to the origin of tetraploid *M. annua*, which is found in central Western Morocco, far from the current distribution of its diploid progenitor *M. annua* [39,61]. Tetraploid *M. annua* is largely monoecious, but rare males have been observed and were sampled in our study here. Unfortunately, we were unable to amplify the male-linked locus in these males, and we thus remain ignorant of the origin of the Y chromosome in these populations.

Our most interesting finding is evidence for the origin of the Y chromosome in hexaploid androdioecious *M. annua* via introgression from one of the more distantly related perennial dioecious species, most probably an ancestor to *M. elliptica* (which is itself hexaploid). This interpretation is convincingly supported by the shared indels in the male-specific locus in both hexaploid *M. annua* and the basal perennial species. Importantly, other than the male-specific locus, we could find no other loci that were contributed by any of the perennial lineages to the *M. annua* hexaploid genome. It would thus seem that hybridization between hexaploid *M. annua* and an ancestor of *M. elliptica* was followed by backcrossing to the annual lineage, with retention of only the sex-determining region carrying the male-specific locus.

The introgression of the sex-determining region of the Y chromosome of hexaploid *M. annua* might have been owing to strong positive selection on the male-determining sequence. Hexaploid *M. annua* occurs as a metapopulation of both monoecious and androdioecious populations [64]. Monoecious individuals have a reproductive advantage over males at low density or during colonization, but males have a siring advantage at high density, so that the Y chromosome quickly rises to intermediate frequencies following immigration [64]. This metapopulation model [65] could also apply to hexaploid *M. annna* at a higher genealogical level. If hexaploid *M. annua* was originally monoecious (with combined sexes maintained by selection for a reproductive assurance because of the possibility of uniparental reproduction at low density), an introgressed male-determining Y chromosome might have been strongly favoured in high-density populations because of the high siring success that male phenotype confers [66,67].

It is also interesting that we could find no evidence for the introgression of other genomic regions into hexaploid *M. annua* from the contributor of its sex-linked region. Hexaploid *M. annua* is an annual species that occupies disturbed habitats such as ploughed fields, roadsides and other ruderal habitats. Its ecological preferences are thus different from those of the perennial species from which its Y chromosome is derived (they are small woody shrubs that occupy less disturbed, often wooded, habitats). If much of their genome had been influenced by selection for their perennial life history, little of it would have been able to persist in the face of negative selection in a frequently disturbed habitat. This possibility would be worth investigating further.

## Data Availability

The Python scripts developed for this study are available on a publicly available GitHub repository under https://github.com/jgerchen/mercurialis_phylogeny. Raw sequencing data are available on the National Center of Biotechnology Information databases (https://www.ncbi.nlm.nih.gov/) under Bioproject PRJNA794737. Additional data are provided in the electronic supplementary material.
